# Comparison of radiation exposure between endoscopic ultrasound‐guided drainage and transpapillary drainage by endoscopic retrograde cholangiopancreatography for pancreatobiliary diseases

**DOI:** 10.1111/den.14060

**Published:** 2021-08-19

**Authors:** Mamoru Takenaka, Makoto Hosono, Madan M. Rehani, Yasutaka Chiba, Rei Ishikawa, Ayana Okamoto, Tomohiro Yamazaki, Atsushi Nakai, Shunsuke Omoto, Kosuke Minaga, Ken Kamata, Kentaro Yamao, Shiro Hayashi, Tsutomu Nishida, Masatoshi Kudo

**Affiliations:** ^1^ Departments of Gastroenterology and Hepatology Kindai University Faculty of Medicine Osaka Japan; ^2^ Department of Radiology Kindai University Faculty of Medicine Osaka Japan; ^3^ Clinical Research Center Kindai University Hospital Osaka Japan; ^4^ Department of Gastroenterology Toyonaka Municipal Hospital Osaka Japan; ^5^ Department of Gastroenterology and Internal Medicine Hayashi Clinic Osaka Japan; ^6^ Global Outreach for Radiation Protection Program Radiation Safety Committee Massachusetts General Hospital Boston USA

**Keywords:** endoscopic retrograde cholangiopancreatography, fluoroscopy, interventional ultrasonography, radiation exposure

## Abstract

**Objectives:**

The transpapillary drainage by endoscopic retrograde cholangiopancreatography (ERCP‐D) cannot be performed without fluoroscopy, and there are many situations in which fluoroscopy is required even in endoscopic ultrasound‐guided drainage (EUS‐D). Previous studies have compared the efficacy, but not the radiation exposure of EUS‐D and ERCP‐D. While radiation exposure in ERCP‐D has been previously evaluated, there is a paucity of information regarding radiation doses in EUS‐D. This study aimed to assess radiation exposure in EUS‐D compared with that in ERCP‐D.

**Methods:**

This retrospective single‐center cohort study included consecutive patients who underwent EUS‐D and ERCP‐D between October 2017 and March 2019. The air kerma (AK, mGy), kerma‐area product (KAP, Gycm^2^), fluoroscopy time (FT, min), and procedure time (PT, min) were assessed. The invasive probability weighting method was used to qualify the comparisons.

**Results:**

We enrolled 372 and 105 patients who underwent ERCP‐D and EUS‐D, respectively. The mean AK, KAP, and FT in the EUS‐D group were higher by 53%, 28%, and 27%, respectively, than those in the ERCP‐D group, whereas PT was shorter by approximately 11% (AK, 135.0 vs. 88.4; KAP, 28.1 vs. 21.9; FT, 20.4 vs. 16.0; PT, 38.7 vs. 43.5). The sub‐analysis limited to biliary drainage cases showed the same trend (AK, 128.3 vs. 90.9; KAP, 27.0 vs. 22.2; FT, 16.4 vs. 16.1; PT, 32.5 vs. 44.4).

**Conclusions:**

This is the first study to assess radiation exposure in EUS‐D compared with that in ERCP‐D. Radiation exposure was significantly higher in EUS‐D than in ERCP‐D, despite the shorter procedure time.

## Introduction

Globally, transpapillary drainage by endoscopic retrograde cholangiopancreatography (ERCP‐D) has been recognized and widely adopted as a treatment for pancreatobiliary diseases,^1^, ^2^, ^3^ and endoscopic ultrasound‐guided drainage (EUS‐D) has gained popularity recently.^4^, ^5^, ^6^, ^7^, ^8^

The common denominator of these procedures is that radiation is essential, and it is well known that there are radiation exposure problems for both the medical personnel and the patient.

Although many studies have compared the usefulness of these techniques, the main comparative parameters used are the success rate of the procedure, clinical success rate, and frequency of complications,^9^, ^10^, ^11^, ^12^, ^13^, ^14^ and no study has used radiation exposure as a comparative parameter.

Radiation exposure accumulates and causes various kinds of damage; however, because the amount of exposure at each time is not so large, it is difficult to understand as an important issue, which is one of the reasons why the awareness of radiation exposure is not high.^15^

While radiation exposure in ERCP‐D has been previously evaluated, there is a paucity of information regarding radiation doses in EUS‐D.

Therefore, we conducted this study to assess radiation exposure in EUS‐D compared with that in ERCP‐D.

## Methods

The study protocol was approved by the Institutional Review Board of Kindai University (IRB No. R02‐121). The study was conducted according to the Declaration of Helsinki as revised in Fortaleza, Brazil, in 2013. All authors had full access to all data of the study and accept responsibility of the submission for publication.

### Patients

This is a single‐center, retrospective study conducted at Kindai University Faculty of Medicine between October 2017 and March 2019. A total of 477 consecutive fluoroscopy‐guided endoscopic procedures (105 EUS‐Ds and 372 ERCP‐Ds) were performed using the same fluoroscopy device with over couch X‐ray tube (CUREVISTA17; Hitachi Co., Tokyo, Japan). Written informed consent was obtained from each patient before the procedure.

### EUS‐D and ERCP‐D

All EUS‐D and ERCP‐D procedures were performed by endoscopists with experience of performing 300 or more procedures annually for at least 5 years. The procedures were performed under conscious sedation using a combination of intravenous propofol and pethidine. For ERCP‐D, a duodenoscope (TJF260V; Olympus Medical Systems, Tokyo, Japan) was advanced into the duodenum, and the transpapillary procedures, including cholangiopancreatography and biliary drainage, were performed under the guidance of fluoroscopy.

For EUS‐D, an echoendoscope (GF‐UCT260; Olympus Medical Systems) was advanced into the stomach or duodenum, and the target of drainage was identified. Next, the target was punctured using a 19‐gauge needle for EUS fine‐needle aspiration (EUS‐FNA), and the contrast medium was injected to obtain a fluoroscopy image of the target. After the insertion of the guidewire into the target through the needle, the puncture sites were dilated using a balloon catheter aided by fluoroscopy. Finally, trans‐gastrointestinal stenting was performed under fluoroscopic guidance. The fluoroscopic system was well maintained with periodic performance assessment by a qualified engineer.

The EUS‐D procedures included EUS‐guided biliary drainage (EUS‐BD) [hepaticogastrostomy (EUS‐HGS)/HGS with antegrade stenting (HGS with AGS)/choledochoduodenostomy (EUS‐CDS)] for dilated bile ducts caused by malignant biliary obstruction (MBO), EUS‐guided gallbladder drainage (EUS‐GBD) for a swollen gallbladder caused by MBO, EUS‐guided cyst drainage (EUS‐CD) for walled‐off necrosis or postoperative pancreatic fistula, and EUS‐guided pancreatic duct drainage (EUS‐PD) for dilated pancreatic duct caused by MBO. EUS‐HGS and HGS with AGS are collectively known as EUS‐HGS‐related procedures (EUS‐HGSR).

In both examinations, fluoroscopy was used only to produce live imaging and essential static images, while other procedures were performed with reference to endoscopic or ultrasound images. In our practice, the main operator inside the fluoroscopy room was a fellow/resident/early‐career endoscopist; the senior professional handled the console outside the fluoroscopy room, controlling pulse selection and other parameters.

### Outcome definitions

The radiation exposure was recorded using standard factors available in most fluoroscopy machines and recommended by the International Commission on Radiological Protection.^16^ These are air kerma (AK, mGy) and kerma‐area product (KAP, Gycm^2^). AK represents the energy emitted when the X‐ray beam from the fluoroscope collides with the air and shows the intensity of the radiation at that point. KAP represents the product of AK and the area of the X‐ray beam in a plane perpendicular to the beam axis.^16^

As a standard practice (U.S. Food and Drug Administration mandate), fluoroscopy machines provide both measurements live during the procedure. Most modern machines display these cumulatively. The details of each procedure, including fluoroscopy time (FT) and procedure time (PT), were recorded in a database that was updated on a per‐study basis.

The primary aim of this study was to compare the values of AK, KAP, FT, and PT between EUS‐D and ERCP‐D.

In a sub‐analysis limited to biliary drainage cases, we performed the same comparison between patients who underwent EUS‐guided biliary drainage (EUS‐HGSR/EUS‐CDS; EUS‐BD group) and those who underwent transpapillary biliary drainage by ERCP (ERCP‐BD group).

The second aim was to compare each EUS‐D procedure in terms of radiation exposure. The values of AK, KAP, FT, and PT were compared for each EUS‐D.

### Statistical analysis

To ensure comparability between the two study groups, confounding variables were adjusted for using the inverse probability weighting (IPW) method,^17^ wherein sex, age, procedure (EUS‐D or ERCP‐D), and disease (pancreatic cancer, biliary cancer, MBO due to cancer elsewhere, or others) were included as the confounders; then, a normalized‐stabilized weight was derived for each patient. With the weight values, box‐whisker plots were created to indicate differences in outcome distributions between the two groups. As the distributions were expected to be skewed, the comparisons between the two groups were made using the method of weighted pairwise comparison^18^, ^19^, ^20^, ^21^ with the above weight, rather than the usual mean comparison. The method of pairwise comparison derives “the net chance of a larger value,” which is the probability that a random patient in the EUS‐D group has a larger value of the outcome than a random patient in the ERCP‐D group, minus the probability of the opposite situation.^20^ For the four outcomes, we estimated the net chance of a larger value and the 95% confidence interval (CI), where CI was derived based on percentiles for the bootstrap distribution with 2000 samples. The net chance of a larger value can be interpreted as the difference between the probability that the potential outcome value if a patient received EUS‐D would be larger than that if a patient received ERCP‐D and the probability of the opposite situation.

## Results

### Baseline characteristics of patients and procedure details

Baseline characteristics of all patients who underwent EUS‐Ds and ERCP‐Ds during this study period and procedure details are shown in Table 1. The mean age was 69 years in the EUS‐D group (76 men and 29 women) and 71 years in the ERCP‐D group (224 men and 148 women). Each procedure type (EUS‐D vs. ERCP‐D) showed heterogeneity in disease.

**Table 1:** Characteristics of the patients and diseases

	Total (*n* = 477)		*P*‐value
	EUS‐D (*n* = 105)	ERCP‐D (*n* = 372)	
Age, years, mean (range)	69 (31–97)	71 (30–97)	0.276
Female sex, *n* (%)	29 (27.6)	148 (39.8)	0.023
Disease, *n* (%)			
Pancreatic cancer	28 (26.7)	57 (15.3)	0.009
Biliary tract cancer	13 (12.3)	59 (15.9)	0.65
MBO due to cancer of other organs	29 (27.6)	30 (8.1)	<0.0001
Others	35 (33.3)	226 (60.8)	<0.0001
CBD stone	1 (0.95)	130 (34.9)	<0.0001
Benign biliary obstruction	1 (0.95)	44 (11.8)	0.0008
Chronic pancreatitis	0 (0)	28 (7.5)	0.0038
Postoperative pancreatic fistula	12 (11.4)	4 (1.1)	<0.0001
WON	14 (13.3)	0 (0)	<0.0001
IPMN	0 (0)	8 (2.2)	0.131
Others	7 (6.7)	12 (3.2)	0.112

*P* < 0.05 was considered statistically significant.

CBD, common bile duct; ERCP‐D, transpapillary drainage by endoscopic retrograde cholangiopancreatography; EUS‐D, endoscopic ultrasound‐guided drainage; IPMN, intraductal papillary mucinous neoplasm; MBO, malignant biliary obstruction; WON, walled‐off necrosis.

For the sub‐analysis limited to biliary drainage cases, Table S1 shows baseline characteristics of all patients who underwent EUS‐BDs and ERCP‐BDs and procedure details (mean age, 73.5 years EUS‐BD group; 74.0 years ERCP‐BD group). Cases of chronic pancreatitis, walled‐off necrosis, and intraductal papillary mucinous neoplasms were excluded. However, the groups still showed heterogeneity in disease.

### Radiation exposure

Table 2 shows the AK, KAP, FT, and PT values in both the EUS‐D and ERCP‐D groups (IPW adjusted, using all data).

**Table 2:** The actual value of radiation exposure (AK, KAP) and FT/PT with IPW adjustment (EUS‐D vs. ERCP‐D)

	EUS‐D (*n* = 105)	ERCP‐D (*n* = 372)	Difference (95% CI)	*P*‐value
AK (mGy) (mean)	135.0	88.4	46.6 (20.4 to 72.8)	0.0005
KAP (Gycm^2^) (mean)	28.1	21.9	6.2 (1.1 to 11.4)	0.0178
FT (min) (mean)	20.4	16.0	4.4 (1.6 to 7.2)	0.0018
PT (min) (mean)	38.7	43.5	−4.8 (−8.7 to −0.9)	0.0156

*P* < 0.05 was considered statistically significant.

AK, air kerma; CI, confidence interval; ERCP‐D, transpapillary drainage by endoscopic retrograde cholangiopancreatography; EUS‐D, endoscopic ultrasound‐guided drainage; FT, fluoroscopy time; IPW, inverse probability weighting; KAP, kerma‐area product; PT, procedure time.

**Figure 1:**
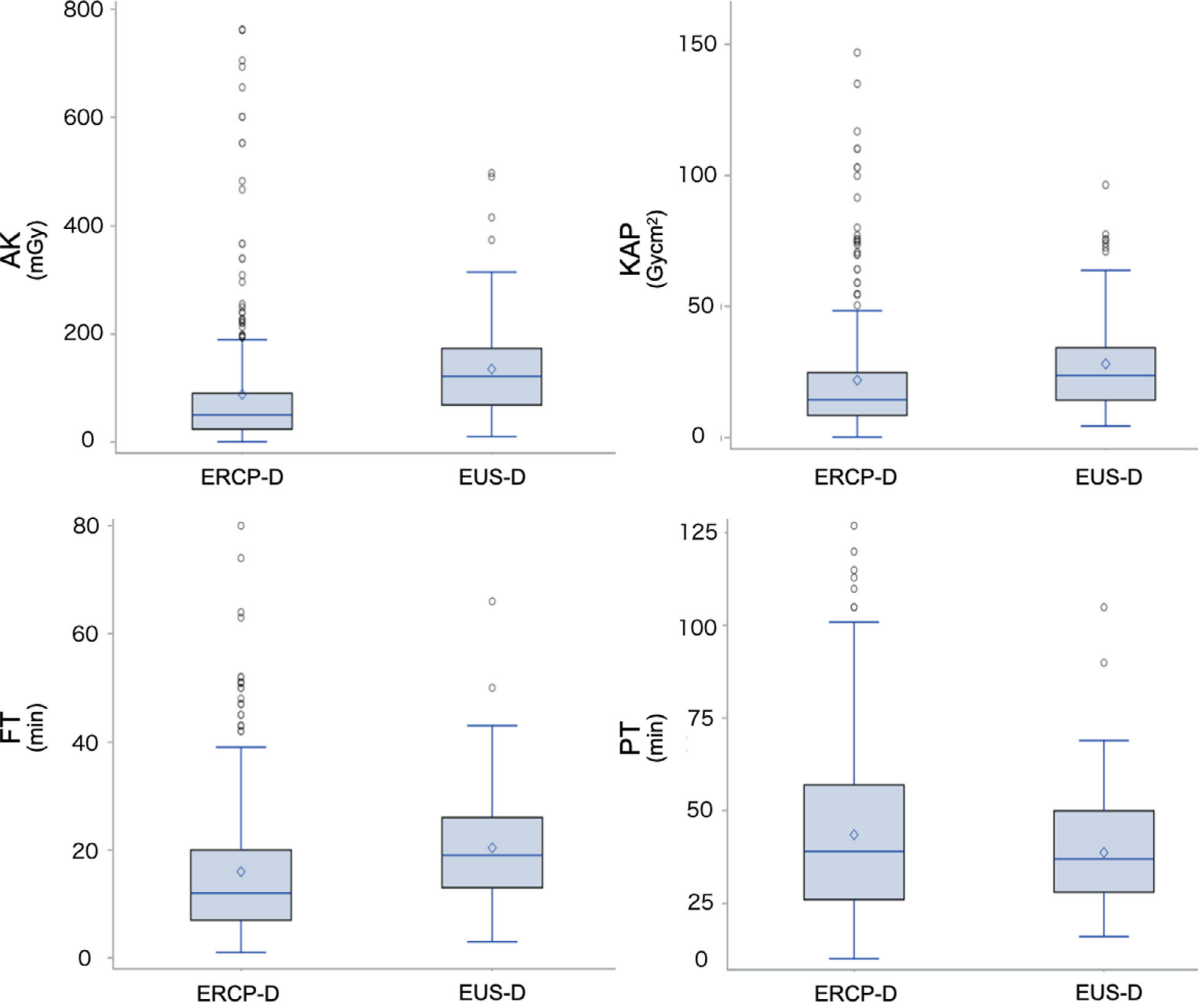
This figure shows the comparison results of IPW adjusted mean value of radiation exposure (AK, KAP), FT, and PT in EUS‐D and ERCP‐D. As shown, there were many outliers in both ERCP‐D and EUS‐D radiation doses. AK, air kerma; ERCP‐D, transpapillary drainage by endoscopic retrograde cholangiopancreatography; EUS‐D, endoscopic ultrasound‐guided drainage; FT, fluoroscopy time; IPW, inverse probability weighting; KAP, kerma‐area product; PT, procedure time.

The mean AK, KAP, and FT in the EUS‐D group were higher by 53%, 28%, and 27%, respectively, than those in the ERCP‐D group, whereas PT was shorter by approximately 11% (AK; 135.0 vs. 88.4, KAP; 28.1 vs. 21.9, FT; 20.4 vs. 16.0, PT; 38.7 vs. 43.5; Fig. 1).

The sub‐analysis limited to biliary drainage cases showed a similar trend. Table 3 shows the AK, KAP, FT, and PT values in both the EUS‐BD and ERCP‐BD groups (IPW adjusted, using all data). In the EUS‐BD group, the mean AK and mean KAP were approximately 41% and 23% higher, respectively, than those in the ERCP‐BD group. The mean FT was similar in both groups. The mean PT was approximately 27% shorter in the EUS‐BD group than that in the ERCP‐BD group (Fig. S1).

**Table 3:** The actual value of radiation exposure (AK, KAP) and FT/PT with IPW adjustment (EUS‐BD vs. ERCP‐BD)

	EUS‐BD (*n* = 66)	ERCP‐BD (*n* = 333)	Difference (95% CI)	*P*‐value
AK (mGy) (mean)	128.3	90.9	37.4 (−14.3 to 72.8)	0.1561
KAP (Gycm^2^) (mean)	27.0	22.2	4.8 (−8.6 to 18.2)	0.4818
FT (min) (mean)	16.4	16.1	0.3 (−6.9 to 7.5)	0.9346
PT (min) (mean)	32.5	44.4	−11.9 (−17.8 to −6.0)	<0.0001

*P* < 0.05 was considered statistically significant.

AK, air kerma; CI, confidence interval; ERCP‐BD, transpapillary biliary drainage by endoscopic retrograde cholangiopancreatography; EUS‐BD, endoscopic ultrasound‐guided biliary drainage; FT, fluoroscopy time; IPW, inverse probability weighting; KAP, kerma‐area product; PT, procedure time.

Table 4 shows the value of radiation exposure parameters (AK, KAP), FT, and PT in each classification of EUS‐D. No significant difference was observed between these groups.

**Table 4:** The actual value of radiation exposure (AK, KAP) and FT/PT in each EUS‐D

	EUS‐HGSR (*n* = 57)	EUS‐CDS (*n* = 9)	EUS‐GBD (*n* = 7)	EUS‐CD (*n* = 30)	EUS‐PD (*n* = 2)
AK (mGy) mean	130.84	160.6	109.2	96.6	164.1
KAP (Gycm^2^) mean	25.5	23.6	20.1	24.0	13.6
FT (min) mean	17.2	17.6	14.8	19.3	28.5
PT (min) mean	33	32	30	42	52

AK, air kerma; CD, cyst drainage; CDS, choledochoduodenostomy; EUS‐D, endoscopic ultrasound‐guided drainage; FT, fluoroscopy time; GBD, gallbladder drainage; HGSR, hepaticogastrostomy related procedures; KAP, kerma‐area product; PD, pancreatic duct drainage; PT, procedure time.

### Net chance estimation

Net chance estimation is a statistical method used to compare two groups with many outliers. As Figure 1 shows, there were many outliers in both ERCP‐D and EUS‐D radiation doses, so this analysis was performed.

The basic concept of net chance estimation is to judge which of the two randomly selected cases from both groups has the larger value. The idea is that after repeating this infinitely, the trend of which of the two groups has the larger value will become apparent.

In Table 5, “*P* (EUS‐D > ERCP‐D)” indicates the probability that a random patient in the EUS‐D group has a larger value of the outcome than a random patient in the ERCP‐D group, whereas “*P* (EUS‐D < ERCP‐D)” indicates the probability of the opposite situation. For example, the result for AK can be interpreted as follows: the probability that the potential AK value would be larger if a patient received EUS‐D than if a patient received ERCP‐D was 73.3%, and the probability of the opposite situation was 26.7%; thus, the former probability was 46.6% higher than the latter. It is evident from Table 5 that the weighted pairwise analysis confirms with statistical significance that “*P* (EUS‐D > ERCP‐D)” is higher than “*P* (EUS‐D < ERCP‐D)” for all three radiation exposure parameters.

**Table 5:** The net chance of larger radiation exposure (AK, KAP) and FT/PT (weighted pairwise comparison) (EUS‐D vs. ERCP‐D)

	*P* (EUS‐D > ERCP‐D)	*P* (EUS‐D < ERCP‐D)	The net chance of larger value (95% CI)	*P*‐value
AK	0.733	0.267	0.466 (0.347 to 0.583)	<0.0001
KAP	0.667	0.333	0.335 (0.200 to 0.465)	<0.0001
FT	0.655	0.319	0.335 (0.200 to 0.470)	<0.0001
PT	0.460	0.523	−0.063 (−0.184 to 0.064)	0.343

*P* < 0.05 was considered statistically significant.

AK, air kerma; CI, confidence interval; ERCP‐D, transpapillary drainage by endoscopic retrograde cholangiopancreatography; EUS‐D, endoscopic ultrasound‐guided drainage; FT, fluoroscopy time; KAP, kerma‐area product; P, probability; PT, procedure time.

## Discussion

This is the first study to assess and reveal radiation exposure in EUS‐D. Radiation exposure was first ever used as a comparison item between EUS‐D and ERCP‐D. Unexpectedly, radiation exposure was found to be significantly higher in EUS‐D than in ERCP‐D, although the PT was shorter in EUS‐D. The sub‐analysis limited to biliary drainage cases showed the same trend. This result may contribute to increase awareness regarding the risk of high radiation exposure among medical personnel and may stimulate better endoscopy development.

Several reasons could be responsible for the higher radiation exposure of EUS‐D: first, the technological barrier exists in both EUS‐D and ERCP‐D. For example, to maintain the position of the guidewire once it is inserted into the target during ERCP‐D, endoscopic visualization of the guidewire can reduce fluoroscopy overuse. Additionally, the wire locking system of the ERCP scope can reduce guidewire mobility and keep the endoscopic visualization intact. However, the guidewire cannot be seen endoscopically in EUS‐D, so full technique of fluoroscopy and scope operation is required. EUS scopes lack EUS‐D‐specific features like guidewire fixation because they were not developed for treatment. Therefore, EUS‐D requires intermittent fluoroscopy to check guidewire position; this increases the accumulated radiation exposure.

Second, the needle used for EUS‐D is a needle developed for EUS‐FNA, which was not intended to be used under fluoroscopy; therefore, its visibility under fluoroscopy is very poor. This also inevitably leads to an increase in cumulative radiation exposure.

Moreover, for live imaging, the fluoroscopic condition is determined by multiplying radiation exposure rate with the “frame rate” (F/R). If the procedure is performed at a high F/R setting, it can be performed using fluoroscopic images with a higher resolution, but the total amount of radiation exposure increases accordingly. In contrast, if the F/R is kept to low, the total amount of radiation exposure can be lowered, but because the resolution of fluoroscopic images is reduced, a proper procedure cannot be performed. Attempts to keep the F/R as low as possible have been reported in the field of cardiology.^22^, ^23^

Even in EUS‐D, F/R can be lowered in certain situations, like post guidewire placement in the bile duct or pancreatic cyst. However, because EUS‐D lacks a dedicated device, it requires high‐quality fluoroscopic images in most situations. Development of dedicated devices may lower radiation exposure during EUS‐D in the future.

Hot Axios (Boston Scientific Corporation, Marlborough, MA, USA) is one of the few devices dedicated to EUS‐D. Many studies have reported Hot Axios' usefulness in EUS‐CD.^24^, ^25^, ^26^ One study reports that EUS‐CD with Hot Axios can minimize fluoroscopy use.^27^ We used fluoroscopy in all EUS‐CD cases to perform internal and external plastic stent placement after Hot Axios placement.

Fluoroscopy time during EUS‐CD with Hot Axios can be speculated to be very short. Therefore, comparing radiation exposure with lumen‐apposing metal stents (Hot Axios) versus that with conventional metal stents in a study limited to EUS‐CD drainage cases would be very important in the future.

In the medical field, radiation is well known to cause harmful effects such as cancer over several years and skin injuries in a relatively shorter period of a few weeks.^28^, ^29^, ^30^, ^31^, ^32^, ^33^, ^34^ The recurrent high‐dose examinations and interventional procedures under fluoroscopy lead the cumulative radiation dose to a range encountered by most atomic‐bomb explosion survivors.^35^, ^36^, ^37^, ^38^

In that sense, ultrasound is the best option under these circumstances, and its use needs to be encouraged. Based on our results, there appears to be an urgent need to develop the best features to make EUS‐D safer.

This study has some limitations. First, the small number of cases in some sub‐groups did not allow detailed analysis. The common features of EUS‐D and ERCP‐D include a wide variety of techniques and the variation in difficulty level between cases. Additionally, a bias exists in the target diseases in both procedures. ERCP‐D is rarely performed for diseases that are eligible for EUS‐GBD/EUS‐CD and vice versa. The background of both groups had many heterogeneities. In the sub‐analysis limited to biliary drainage cases, these heterogeneities were slightly reduced. However, outliers existed, especially in the ERCP‐BD group due to the large variability in cases. Although the propensity score analysis using the IPW method minimized bias, we still recommend that studies be conducted with the same disease or procedure for comparison. Hence, we performed a net chance estimation for our study. Even with net chance estimation, the probability that EUS‐D has a higher radiation exposure than ERCP‐D is significantly high, further strengthening our result's credibility.

Second, this was a single‐center study using one type of fluoroscopy equipment. A better‐equipped study may yield information for generalization of outcomes and help in providing further details regarding this topic.

Third, we excluded cases of balloon enteroscopy‐assisted ERCP (BE‐ERCP). BE‐ERCP is an important option for biliary drainage. However, BE‐ERCP requires time to reach the papilla or bile duct jejunal anastomosis using a double or single balloon endoscope before the drainage treatment. Fluoroscopy is also required. These factors are related to scope insertion, not biliary drainage, the focus of this study. Therefore, we excluded these cases from the study. Comparison of radiation exposure between conventional ERCP and BE‐ERCP would need to be studied separately in a larger number of cases.

In conclusion, higher radiation exposure in EUS‐D than in ERCP‐D, despite the shorter PT, is of significance for the future design of equipment for modifying instrument operation and technique and for identifying a selection strategy for the modality. There is a clear need for awareness among medical personnel dealing with endoscopic procedures to minimize radiation risks to patients and staff.

## Conflict of Interest

Authors declare no conflict of interest for this article.

## Funding Information

None.

## Supporting information


**Figure S1** This figure shows the comparison results of IPW adjusted mean value of radiation exposure (AK, KAP), FT, and PT in EUS‐BD and ERCP‐BD. AK, air kerma; ERCP‐BD, transpapillary biliary drainage by endoscopic retrograde cholangiopancreatography; EUS‐BD, endoscopic ultrasound‐guided biliary drainage; FT, fluoroscopy time; IPW, inverse probability weighting; KAP, kerma‐area product; PT, procedure time.

**Table S1** This table shows baseline characteristics of all patients who underwent EUS‐BDs and ERCP‐BDs and procedure details.
