# Supplementary figures and images for: Comparison of radiation exposure between endoscopic ultrasound‐guided drainage and transpapillary drainage by endoscopic retrograde cholangiopancreatography for pancreatobiliary diseases
Source: Dig Endosc. 2021 Aug 19;34(3):579–86. doi: 10.1111/den.14060 (PMC9292288; doi:10.1111/den.14060)

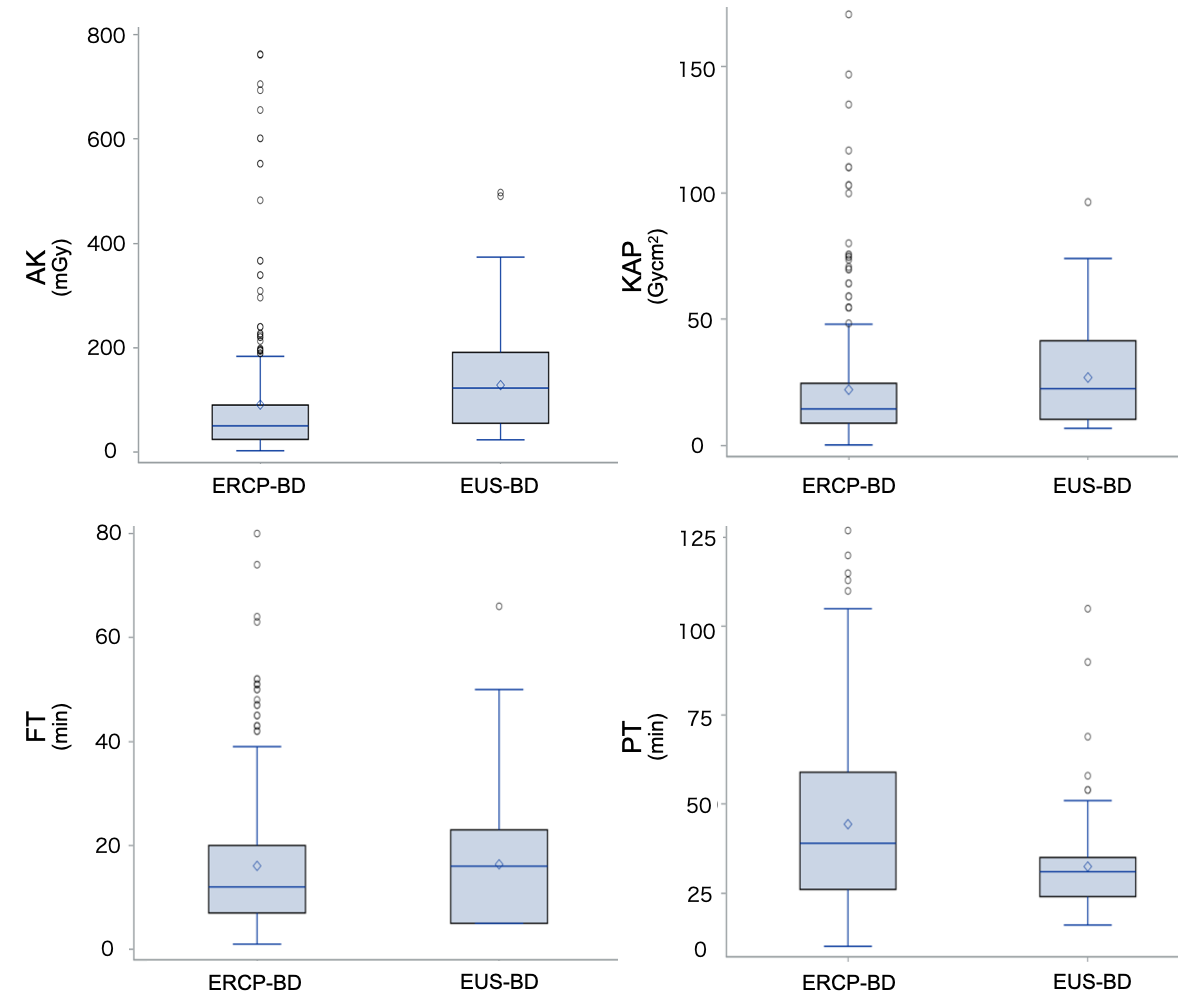

Supplement: Supplementary file 1 — Figure S1 This figure shows the comparison results of IPW adjusted mean value of radiation exposure (AK, KAP), FT, and PT in EUS‐BD and ERCP‐BD. AK, air kerma; ERCP‐BD, transpapillary biliary drainage by endoscopic retrograde cholangiopancreatography; EUS‐BD, endoscopic ultrasound‐guided biliary drainage; FT, fluoroscopy time; IPW, inverse probability weighting; KAP, kerma‐area product; PT, procedure time. [file DEN-34-579-s002.tif]
