# Supplementary material for: Comparison of radiation exposure between endoscopic ultrasound‐guided drainage and transpapillary drainage by endoscopic retrograde cholangiopancreatography for pancreatobiliary diseases
Source: Dig Endosc. 2021 Aug 19;34(3):579–86. doi: 10.1111/den.14060 (PMC9292288; doi:10.1111/den.14060)
Supplement: Supplementary file 2 — Table S1 This table shows baseline characteristics of all patients who underwent EUS‐BDs and ERCP‐BDs and procedure details. [file DEN-34-579-s001.docx]

**Table S1. Characteristics of patients and diseases (biliary drainage cases only)**

|  | Total (*n* = 399) | | *P* value |
| --- | --- | --- | --- |
|  | EUS-BD  (*n* = 66) | ERCP-BD  (*n* = 333) |  |
| Age (mean, years)  Range | 73.5  (40–86) | 74.0  (30–86) | 0.780 |
| Female sex, *n* (%) | 20 (30.3) | 134 (40.2) | 0.166 |
| Disease, *n* (%) |  |  |  |
| Pancreatic cancer | 28 (42.4) | 56 (16.8) | < 0.0001 |
| Biliary tract cancer | 8 (12.1) | 59 (17.7) | 0.37 |
| MBO due to cancer of other organs | 29 (43.9) | 30 (9.0) | < 0.0001 |
| Others | 1 (1.5) | 188 (56.5) | < 0.0001 |
| CBD stone | 0 (0) | 130 (39.0) |  |
| Benign biliary obstruction | 1 (1.5) | 42 (12.6) |  |
| Chronic pancreatitis | 0 (0) | 0 (0) |  |
| Postoperative pancreatic fistula | 0 (0) | 4 (1.2) |  |
| WON | 0 (0) | 0 (0) |  |
| IPMN | 0 (0) | 0 (0) |  |
| Others | 0 (0) | 12 (3.6) |  |

*P* < 0.05 was considered statistically significant.

EUS-BD: endoscopic ultrasound-guided biliary drainage, ERCP-BD: transpapillary biliary drainage by endoscopic retrograde cholangiopancreatography, *n*: number, MBO: malignant biliary obstruction, CBD: common bile duct, WON: walled-off necrosis, IPMN: intraductal papillary mucinous neoplasm.
